# Prevalence of Radiographic Alveolar Bone Loss as a Function of Age in the Periodontics Clinic Population at the College of Dentistry, King Saud University, Riyadh, Saudi Arabia

**DOI:** 10.3290/j.ohpd.b4424899

**Published:** 2023-09-22

**Authors:** Mansour Alaskar, Hani S. AlMoharib, Amani Mirdad, Arwa A. Talakey, Paul Levi, Abdulmonem Alshihri

**Affiliations:** a Professor and Periodontist, Department of Periodontics and Community Dentistry, College of Dentistry, King Saud University, Riyadh, Saudi Arabia. Study design, patient examination, data collection, wrote the manuscript.; b Assistant Professor and Periodontist, Department of Periodontics and Community Dentistry, College of Dentistry, King Saud University, Riyadh, Saudi Arabia. Patient examination, data collection, statistical analysis.; c Assistant Professor and Periodontist, Department of Periodontics and Community Dentistry, College of Dentistry, King Saud University, Riyadh, Saudi Arabia. Patient examination, data collection, reviewed the manuscript.; d Assistant Professor, Department of Periodontics and Community Dentistry, College of Dentistry, King Saud University, Riyadh, Saudi Arabia. Patient examination, data collection.; e Clinical Associate Professor, Department of Periodontology, Tufts University School of Dental Medicine, Boston, MA, USA. Wrote the proposal, edited the manuscript, designed methodology.; f Associate Professor and Prosthodontist, Department of Prosthetic Dental Science, College of Dentistry, King Saud University, Riyadh, Saudi Arabia. Statistical analysis, edited and reviewed the manuscript.

**Keywords:** alveolar bone loss, diabetes mellitus, hypertension, obesity, osteoporosis, prevalence

## Abstract

**Purpose:**

The purpose of the present observational study was to assess the prevalence of radiographic alveolar bone loss (ABL) as a function of age at the Periodontics Clinics at the College of Dentistry, King Saud University, Riyadh, Saudi Arabia.

**Materials and Methods:**

Medical and dental healthcare records of individuals visiting the Periodontics Clinics at College of Dentistry, King Saud University, Riyadh Saudi Arabia were assessed. The following information was retrieved: age, gender, educational status, and systemic diseases (diabetes mellitus [DM], hypertension, osteoporosis and obesity). Digital full-mouth radiographs were retrieved from patients’ dental records, and marginal bone loss (MBL) was assessed on the mesial and distal surfaces of all teeth. Logistic regression analyses (LRA) were done to assess the correlation between ABL and gender, age, educational status and duration since diagnosis of the aforementioned systemic conditions. p < 0.05 was considered statistically significant.

**Results:**

In total, medical and periodontal healthcare records of 495 individuals were retrieved and assessed. All individuals were citizens of the KSA. Among these, 107 were healthy controls and 98, 95, 96 and 99 individuals had a medical diagnosis of type-2 DM, hypertension, obesity and osteoporosis, respectively. There was no statistically significant difference in the mean age and gender of all medically compromised participants. The prevalence of mild, moderate, and severe periodontitis in the total patient population was 51.4%, 37.5% and 36.5%, respectively. Among all healthy controls, the prevalence of mild, moderate, and severe periodontitis was 16.3%, 25.5% and 33.4%, respectively. There was no difference in the prevalence of mild, moderate, and severe periodontitis in relation to advancing age in the entire patient population.

**Conclusion:**

Advancing age did not seem to affect ABL in the present patient population. Patient education, oral hygiene maintenance and SES seem to be more predictable indicators of ABL than increasing age.

Periodontal disease initially manifests as gingivitis, which is limited to inflammation of soft tissues.^1^ However, if left undiagnosed and not treated in a timely manner, the severity of periodontal inflammation increases and enhances the risk of alveolar bone loss (ABL) around teeth (periodontitis). The undesirable yet preventable outcome of ABL is loss of dentition.^1^ Risk factors that can predispose vulnerable patient populations to periodontitis can broadly be categorised into local or systemic risk factors. From a local perspective, the most common risk factor of periodontitis is poor oral hygiene maintenance.^26^ In other words, irregular and inadequate toothbrushing and flossing of interproximal spaces increases the risk of oral inflammatory conditions, including periodontitis.^26^ According to Haran and McCormick,^15^ imbalances in gut microbes and immune function elevates the risk of diseases in aged populations compared with younger individuals. From an oral heath point of view, it has been reported that periodontal probing depth (PD) and alveolar bone loss (ABL) are worse in individuals over 60 years of age compared with younger individuals (< 45 years of age).^8,11,18,21^

The global prevalence of DM is increasing and the Kingdom of Saudi Arabia (KSA) is listed among the “top ten countries” with the highest prevalence of DM (approximately 24%).^20^ A worrying epidemiological observation is that the diagnosis of DM has increased nearly 10-fold in the KSA in recent years.^4^ It has also been reported that nearly 14% of the healthcare budget in the KSA is spent on the management of DM.^23^ Likewise, in the KSA, the prevalences of other debilitating health conditions such as hypertension, obesity and osteoporosis are 32.35%, 35% and nearly 60%, respectively.^2,24,25^ These systemic diseases (DM, obesity, osteoporosis and hypertension) are also well-known risk factors of periodontitis.^14,19,30^ Al-Zahrani and Kayal^3^ assessed ABL among patients from a dental school in the Western province of the KSA. The results showed that approximately 68% of the 282 individualsf assessed had ABL, which was more often manifested in patients with systemic diseases such as DM.^3^ Here, it is also worth mentioning that advancing age is also directly associated with occurrence of various systemic conditions, including those described above.^13^ To the authors’ knowledge from pertinent indexed literature, the relationship between age and periodontitis in populations with systemic diseases has been inadequately explored.

With this background, the purpose of the present observational study was to assess the prevalence of radiographic ABL as a function of age at the Periodontics Clinics at College of Dentistry, King Saud University. The study hypothesis was that there is no relationship between ABL and age in vulnerable patient populations.

## Materials and Methods

### Ethics Approval

The study protocol (Project # E-18-3099) was reviewed and approved by the Institutional Review Board of the Health Sciences Colleges Research on Human Subjects, College of Medicine, King Saud University, Riyadh, Saudi Arabia (Approval # 18/0284/IRB). Participation was voluntary; it was mandatory for all participants to read and sign a written informed consent form prior to having their healthcare records assessed. There were no penalties and/or consequences for individuals who declined to have their healthcare records assessed and/or withdrew at any stage of investigation. All volunteers were informed that their personal information, including name, home/office addresses, photos, and other contact details (such as phone numbers and email addresses) would be kept confidential. Consenting individuals were requested to return a signed version of the consent form to the department of Periodontology at King Saud University in a pre-stamped envelope.

### Study Location

The present retrospective observational study was performed at the Department of Periodontics and Community Dentistry, College of Dentistry, King Saud University, Riyadh, KSA between November 2018 and January 2022.

### Study Participants

Digital healthcare records of individuals visiting the Periodontics Clinics at the Department of Periodontics and Community Dentistry at the College of Dentistry, King Saud University, were assessed in the present study.

### Inclusion and Exclusion Criteria

Individuals (KSA citizens) who agreed to their medical and periodontal healthcare records being evaluated were included in the present study. Individuals habitually using combustible and/or non-combustible nicotinic products were not included. Third molars, grossly carious and supernumerary teeth, as well as records of pregnant/lactating patients were not assessed.

### Evaluation of Medical Records

Patients’ digital healthcare records were assessed by one investigator and the following information was retrieved: age, gender, educational status, and identification of systemic diseases (DM, hypertension, osteoporosis and obesity).

### Assessment of Digital Full-mouth Radiographs

Digital full-mouth radiographs were retrieved from patients’ dental records, and marginal bone loss (MBL) was assessed on the mesial and distal surfaces of all teeth. MBL was measured in millimeters (mm) as the vertical distance from 2 mm below the cementoenamel junction to the alveolar crest.^18^ These measurements were performed by one blinded, trained and calibrated investigator (Kappa score 0.88).

### Classification of Periodontitis

In the present study, patients with MBL between 2 and 3 mm were categorised as having mild periodontitis.^16^ Individuals with an MBL ranging between 3 and 4 mm or >4 mm were categorised as having moderate or severe periodontitis, respectively.^16^

### Age Distribution

Patients were grouped by age in years as follows: (a) 20 to 29.9; (b) 30 to 39.9; (b) 40 to 49.9; (c) 50 to 59.9; (d) 60 to 69.9; and (e) over 70.

### Sample-size Estimation

Power analysis was done on pilot data using a software program (nQuery Advisor 6.0, Statistical Solutions; Saugas, MA, USA) with an effect size and alpha of 0.30 and 0.05, respectively. Sample size estimation was based on the study by Eke et al,^10,11^ in which the odds of developing periodontitis were 2.3 when comparing individuals aged 35 to 47 years with those 30 to 34 years old. Assuming a mean difference of 2 mm in mesial and distal MBL among individuals aged 20-29.9 years old and those over 49.9 years, it was estimated that a sample size of at least 450 individuals would achieve 95% study power with an alpha of 5%.

### Statistical Analysis

Statistical analyses were done by a trained statistician who was blinded to the study groups. SPSS version 20 (IBM; Armonk, NY, USA) was used for statistical analyses. Group comparisons were done using the Mann-Whitney U- and paired t-tests. Normality of data was assessed using the Kolmogorov-Smirnov and the Shapiro-Wilk tests. Logistic regression analyses were done to assess the correlation between ABL and gender, age, hypertension, obesity, DM, and educational status. p-values < 0.05 were considered statistically significant.

## Results

### Characteristics of the Population

In total, medical and periodontal healthcare records of 495 individuals were retrieved and assessed. All individuals were citizens of the KSA. Of these, 107 were healthy controls, and 98, 95, 96 and 99 individuals had a medical diagnosis of type-2 DM, hypertension, obesity and osteoporosis, respectively. There was no statistically significant difference in the mean age and gender of all medically compromised participants. Among the systemically healthy controls, the number of male participants was statistically significantly higher than females (p < 0.05). In the entire population, 16.4% and 57.7% patients reported to have school- and university-level education, respectively (p < 0.05). Of the healthy controls, the majority had attained a university-level education (86.9%). There was no difference in the educational status among patients with type-2 DM, hypertension and obesity (Table 1). Table 2 demonstrates the age-wise distribution of systemically healthy and medically-compromised individuals.

**Table 1:** Characteristics of the study groups

Parameters	All individuals	Type-2 diabetes mellitus	Hypertension	Obesity	Osteoporosis	Healthy controls
Number of individuals (n)	495 (100%)	98 (19.8%)	95 (19.2%)	96 (19.4%)	99 (20%)	107 (21.6%)
Males (%)	294 (59.4%)	54 (55.1%)	50 (52.6%)	57 (59.4%)	31 (31.3%)	102 (94.4%)^‡^
Females (%)	201 (40.6%)	44 (44.9%)	45 (47.4%)	39 (40.6%)	68 (68.7%)	5 (5.6%)
Mean age	55.4 ± 4.7 years	52.7 ± 2.8 years	62.4 ± 6.7 years	57.1 ± 2.3 years	66.6 ± 10.5 years	65.8 ± 12.5 years
Education level						
School-level	81 (16.4%)*^†^	23 (23.5%)	19 (20%)	22 (22.9%)	15 (15.2%)*^†^	2 (1.9%)*^†^
College-level	128 (25.9%)^†^	36 (36.7%)	25 (26.3%)	37 (38.5%)	18 (18.2%)^†^	12 (11.2%)^†^
University-level	286 (57.7%)	39 (39.8%)	51 (53.7%)	37 (38.6%)	66 (66.6%)	93 (86.9%)

*Compared with college level education in all individuals (p < 0.05). ^†^Compared with university-level education in all individuals (p < 0.05).

**Table 2:** Number of participants in relation to advancing age

Age groups in years	All individuals	Type-2 diabetes mellitus	Hypertension	Obesity	Osteoporosis	Healthy controls
20 to 29.9	26 (5.2%)	0	0	6 (6.3%)	0	20 (18.7%)
30 to 39.9	43 (8.7%)	4 (4.1%)	3 (3.2%)	15 (15.6%)	0	21 (19.6%)
40 to 49.9	116 (23.4%)	36 (36.7%)	24 (25.7%)	31 (32.3%)	0	25 (23.4%)
50 to 59.9	165 (33.3%)	32 (32.6%)	38 (40%)	21 (21.9%)	53 (53.5%)	21 (19.6%)
60 to 69.9	73 (14.7%)	14 (14.3%)	17 (17.9%)	11 (11.4%)	20 (20.2%)	11 (10.3%)
>70	72 (14.7%)	12 (12.3%)	13 (13.2%)	12 (12.5%)	26 (26.3%)	9 (8.4%)
All individuals	495	98	95	96	99	107

### Prevalence of Periodontitis

The prevalence of mild, moderate and severe periodontitis in the total patient population was 51.4%, 37.5% and 36.5%, respectively. Among all healthy controls, the prevalence of mild, moderate and severe periodontitis was 16.3%, 25.5% and 33.4% respectively. There was no difference in the prevalence of mild, moderate and severe periodontitis in relation to advancing age in the entire patient population. These results are shown in Table 3.

**Table 3:** Prevalence of periodontitis (percentages) in relation to age (in years)

Study population	Severity of periodontitis								
	n	Mild	SE	Moderate	SE	Severe	SE	Mean MBL*	SE
Total population	495 (100%)	51.4	2.7	37.5	1.8	36.5	1.06	2.7	0.4
All healthy controls	107 (100%)	16.3%	2.3	25.5	2.05	33.4	1.7	1.6	0.13
20 to 29.9	20 (18.7%)	0.0	0.0	0.0	0.0	0.0	0.0	0.3	0.003
30 to 39.9	21 (19.6%)	19.04%	0.0	0.0	0.0	0.0	0.0	2.2	0.08
40 to 49.9	25 (23.4%)	24%	4.7	16.7%	3.5	0.0	0.0	1.8	0.07
50 to 59.9	21 (19.6%)	9.5%	1.8	33.3%	2.2	19.04%	2.4	2.3	0.18
60 to 69.9	11 (10.3%)	18.2%	0.07	36.4%	0.14	18.2%	0.08	2.04	0.11
>70	9 (8.4%)	11.1%	0.08	33.3	0.07	55.5%	0.09	3.08	0.2
Diabetic patients	98 (100%)	20.8	1.7	51.6	1.5	50.6	1.4	3.05	0.11
20 to 29.9	0	0.0	0.0	0.0	0.0	0.0	0.0	0.0	0.0
30 to 39.9	4 (4.1%)	0.0	0.0	0.0	0.0	0.0	0.0	0.0	0.0
40 to 49.9	36 (36.7%)	27.7	2.1	47.7%	1.8	13.9%	1.2	2.1	0.07
50 to 59.9	32 (32.6%)	15.6%	1.4	59.4%	1.4	28.1	1.5	2.5	0.06
60 to 69.9	14 (14.3%)	14.3%	1.8	50%	1.4	57.1%	0.09	2.9	0.04
>70	12 (12.3%)	0.0	0.0	58.3	1.3	41.7	1.2	3.3	0.08
Hypertension	95	50.6%	3.7	55.1%	2.2	46.3%	2.8	3.5	1.8
20 to 29.9	0	0.0	0.0	0.0	0.0	0.0	0.0	0.0	0.0
30 to 39.9	3 (3.2%)	33.3%	1.8	66.7%	0.05	0.0	0.0	1.7	0.05
40 to 49.9	24 (25.7%)	16.7%	1.5	62.5%	2.1	20.8%	2.3	2.4	1.8
50 to 59.9	38 (40%)	86.8%	1.4	13.2%	1.7	0.0	0.0	2.5	1.04
60 to 69.9	17 (17.9%)	0.0	0.0	70.6%	2.2	29.4%	1.7	2.8	0.7
>70	13 (13.2%)	0.0	0.0	46.2%	1.5	53.8%	1.5	2.7	0.5
Obesity	96	77.1%	2.5	23.1%	1.4	0.0	0.0	2.4	0.8
20 to 29.9	6 (6.3%)	83.3%	1.8	19.7	0.8	0.0	0.0	2.3	1.2
30 to 39.9	15 (15.6%)	73.3%	2.7	26.7%	0.0	0.0	0.0	2.6	1.5
40 to 49.9	31 (32.3%)	0.0	0.0	0.0	0.0	0.0	0.0	0.0	0.0
50 to 59.9	21 (21.9%)	0.0	0.0	0.0	0.0	0.0	0.0	0.0	0.0
60 to 69.9	11 (11.4%)	0.0	0.0	0.0	0.0	0.0	0.0	0.0	0.0
>70	12 (12.5%)	0.0	0.0	0.0	0.0	0.0	0.0	0.0	0.0
Osteoporosis	99	0.0	0.0	53.5%	3.7	49.6	2.1	3.5	0.7
20 to 29.9	0	0.0	0.0	0.0	0.0	0.0	0.0	0.0	0.0
30 to 39.9	0	0.0	0.0	0.0	0.0	0.0	0.0	0.0	0.0
40 to 49.9	0	0.0	0.0	0.0	0.0	0.0	0.0	0.0	0.0
50 to 59.9	53 (53.5%)	0.0	0.0	49.1%	2.9	50.9%	3.1	3.7	1.4
60 to 69.9	20 (20.2%)	0.0	0.0	55%	3.4	45%	2.2	3.2	1.8
>70	26 (26.3%)	0.0	0.0	57.7%	2.8	42.3%	2.5	3.6	1.2

MBL: Marginal bone loss in millimeters. *Data are presented as the means of mesial and distal MBL. Data are presented as means ± standard error.

### Regression Analyses

Logistic regression analyses showed no statistically significant correlation between ABL and HbA1c, gender, age, educational status and duration since diagnosis of systemic conditions (data not shown).

## Discussion

In the present observational cohort study, it was speculated that advancing age is an important factor that influences the severity of ABL (periodontitis). This speculation was based on results from a previous case-control study,^18^ in which individuals aged over 60 years had more severe periodontal inflammation compared with younger individuals < 49 years old. Moreover, there is sufficient scientific evidence showing that the ABL is higher in immunosuppressed individuals than in controls (medically healthy individuals).^12,30^ In this context, we categorised the patient population into medically-challenged and healthy groups, and further categorised these individuals into six age groups (Table 2). It was anticipated that medically-challenged and healthy individuals aged ≥ 60 years would demonstrate higher mean ABL compared with younger counterparts (< 30 years old). From an experimental standpoint, Wu et al^29^ assessed the influence of advancing age on challenge by oral pathogens and resultant host response as well as osteoclast numbers in young vs aged mice.^29^ The results showed that bacterial diversity was reduced in aged mice. In other words, colonisation of periodontopathogenic bacteria (*Porphyromonas gingivalis* [*P. gingivalis*]) was significantly higher in older than younger mice.^29^ In that study on animal models, increased gingival *P. gingivalis* colonization was associated with an enhanced osteoclastic function as well as serum tumor necrosis-factor alpha levels. Several factors make the present investigation unique in contrast to a previous study^16^ from theh USA with similar objectives. In the study by Helmi et al,^16^ tobacco smokers and individuals with a variety of geographic backgrounds were enrolled. However, in the present study, stringent eligibility criteria were imposed. Firstly, we performed the present study exclusively among self-reported non-smokers and patients from a specific geographic background, that is, KSA. One reason for this is that habitual use of nicotinic products and demographic variables are known to impact the prevalence of periodontitis.^5,7,10^ It is worth remembering that habitual use of combustible nicotinic products is associated with an increased formation and accumulation of advanced glycation endproducts and destructive inflammatory cytokines in periodontal tissues,^5,7,10,17,22^ which enhance the progression of periodontal inflammatory conditions, including periodontitis. From the authors’ perspective, application of such stringent standards facilitates meaningful comparison of results from individuals belonging to a specific ethnicity and/or geographic location.

Surprisingly, the present results showed no statistically significant relationship between advancing age and increased prevalence of ABL in medically and systemically healthy controls. Several factors seem to have contributed towards such an outcome. The most common and well-known risk factor of increased ABL is poor routine oral hygiene maintenance.^28^ Moreover, it has also been reported that a higher education and socioeconomic (SES) is associated with better health status, including oral health, in contrast to those with less education and a lower SES. It is notable that we found no statistically significant difference in the prevalence of ABL between younger and older patients with type-2 DM. It is well established that under optimal glycemic control, diabetic individuals can have clinical-radiographic statuses similar to non-diabetic individuals.^18^ Similarly, these factors also contribute towards a stable glycemic status in patients with DM.^28^

There are several limitations associated with the results reported in the present investigation. First, it is worth mentioning that the primary objective of the current investigation was to assess the prevalence of ABL as a measure of advancing age in systemically healthy and medically-compromised individuals. In this context, clinical parameters such as periodontal soft tissue inflammation (including clinical attachment loss, bleeding on probing and probing depth) were not assessed. Moreover, calibration of the investigators who initially took the bitewing radiographs was impractical. Therefore, it is possible that ABL levels might have differed from those taken from radiographic examinations done by calibrated assessors. Also, it could not be verified whether or not a standard technique (such as the long-cone paralleling method) or calibration of individuals taking the radiographs had been performed. Furthermore, a major limitation of two-dimensional radiographs is that they are unable to detect furcation and/or crater involvements in bone.^27^ Additional studies based on an advanced imaging technique such as cone-beam computed tomography may help to precisely evaluate patterns of ABL in medically-challenged and systemically healthy populations exposed to oral inflammatory diseases, including periodontitis. There is sufficient evidence in the indexed literature to confirm that habitual use of nicotinic products increases the risk of periodontitis in susceptible patients,^7,11^ that this relationship is independent of individuals’ systemic health status.^7,11^ In this study, nicotinic product users were not assessed, as this variable could have potentially biased the prevalence-related outcomes. It is speculated that the prevalence of periodontitis is higher in medically-compromised tobacco smokers and smokeless-tobacco users than medically-challenged and systemically healthy individuals who do not use any form of nicotinic products. Moreover, it is well-known that lower income status is a risk-factor of periodontitis.^6,9,18^ Regrettably, the records evaluated here did not contain any information related to SES of patients. It is strongly recommended that community-based health awareness programs should routinely be conducted to educate the general public about the connection between oral and systemic health, as well as the importance of routine oral hygiene maintenance on overall health.

## Conclusion

Advancing age did not seem to affect ABL in the present patient population. Patient education, oral hygiene maintenance and SES seem to be more predictable indicators of ABL than increasing age.
